# Association of Metformin with Susceptibility to COVID-19 in People with Type 2 Diabetes

**DOI:** 10.1210/clinem/dgab067

**Published:** 2021-02-09

**Authors:** Jingya Wang, Jennifer M Cooper, Krishna Gokhale, Dionisio Acosta-Mena, Samir Dhalla, Nathan Byne, Joht Singh Chandan, Astha Anand, Kelvin Okoth, Anuradhaa Subramanian, Mansoor N Bangash, Thomas Jackson, Dawit Zemedikun, Tom Taverner, Wasim Hanif, Sandip Ghosh, Parth Narendran, Konstantinos A Toulis, Abd A Tahrani, Rajendra Surenthirakumaran, Nicola J Adderley, Shamil Haroon, Kamlesh Khunti, Christopher Sainsbury, G Neil Thomas, Krishnarajah Nirantharakumar

**Affiliations:** 1 Institute of Applied Health Research, University of Birmingham, Birmingham, UK; 2 Cegedim Health Data, Cegedim Rx, London, UK; 3 The Health Improvement Network (THIN), London, UK; 4 Institute of Clinical Sciences, University of Birmingham, Birmingham, UK; 5 Department of Critical Care, University Hospitals Birmingham NHS Foundation Trust, Birmingham, UK; 6 Institute of Inflammation and Ageing, University of Birmingham, Birmingham, UK; 7 Department of Diabetes and Endocrinology, University Hospitals Birmingham NHS Foundation Trust, Birmingham, UK; 8 Institute of Immunology and Immunotherapy, University of Birmingham, Birmingham, UK; 9 Institute of Metabolism and Systems Research, University of Birmingham, Birmingham, UK; 10 Centre for Endocrinology, Diabetes and Metabolism (CEDAM), Birmingham Health Partners, Birmingham, UK; 11 Department of Community and Family Medicine, Faculty of Medicine, University of Jaffna, Jaffna, Sri Lanka; 12 Diabetes Research Centre, University of Leicester, Leicester; 13 Department of Diabetes, Gartnavel General Hospital, NHS Greater Glasgow and Clyde, Glasgow, UK; 14 Midlands Health Data Research UK, Birmingham, UK

**Keywords:** type 2 diabetes mellitus, metformin, COVID-19, SARS-CoV-2 infection

## Abstract

**Objective:**

Diabetes has emerged as an important risk factor for mortality from COVID-19. Metformin, the most commonly prescribed glucose-lowering agent, has been proposed to influence susceptibility to and outcomes of COVID-19 via multiple mechanisms. We investigated whether, in patients with diabetes, metformin is associated with susceptibility to COVID-19 and its outcomes.

**Research Design and Methods:**

We performed a propensity score–matched cohort study with active comparators using a large UK primary care dataset. Adults with type 2 diabetes patients and a current prescription for metformin and other glucose-lowering agents (MF+) were compared to those with a current prescription for glucose-lowering agents that did not include metformin (MF−). Outcomes were confirmed COVID-19, suspected/confirmed COVID-19, and associated mortality. A negative control outcome analysis (back pain) was also performed.

**Results:**

There were 29 558 and 10 271 patients in the MF+ and MF− groups, respectively, who met the inclusion criteria. In the propensity score–matched analysis, the adjusted hazard ratios for suspected/confirmed COVID-19, confirmed COVID-19, and COVID-19-related mortality were 0.85 (95% CI 0.67, 1.08), 0.80 (95% CI 0.49, 1.30), and 0.87 (95% CI 0.34, 2.20) respectively. The negative outcome control analysis did not suggest unobserved confounding.

**Conclusion:**

Current prescription of metformin was not associated with the risk of COVID-19 or COVID-19-related mortality. It is safe to continue prescribing metformin to improve glycemic control in patients with.

A novel strain of coronavirus, severe acute respiratory syndrome coronavirus 2 (SARS-CoV-2) has spread swiftly across countries and continents and become a global pandemic, crippling health systems and economies. This infection was often found to cause a severe multisystem illness with increased severity particularly in older adults and those with comorbidities, including obesity (1). As of February 21, 2021, COVID-19 was estimated to have infected over 111 million people and caused more than 2.4 million deaths (2). Both type 1 and type 2 diabetes have been recognized as significant risk factors for adverse outcomes and associated mortality from COVID-19 (3, 4). On the other hand, glucose-lowering medications commonly prescribed in diabetes are also known to have modulatory effects on inflammation (5). The potential impact of these therapeutic agents on susceptibility to COVID-19 is therefore of significant clinical and public health interest (6, 7).

A number of retrospective cohort and observational studies have suggested that concomitant use of metformin is associated with beneficial clinical outcome in respiratory diseases including tuberculosis (8), chronic obstructive pulmonary disease (9), pneumonia (10), and asthma (11). Furthermore, metanalysis suggests pre-admission use of metformin improves septic shock outcomes in patients with diabetes (12). Metformin has been associated with a reduction in CVD in a subgroup of patients with obesity and is the recommended first-line therapy in type 2 diabetes (13). There is therefore an urgent need to clarify whether metformin influences susceptibility to COVID-19. Two recent meta-analyses of observational studies have suggested reduced mortality from COVID-19 in patients taking metformin (14, 15). These studies only included patients who were hospitalized with COVID-19, and they do not address the important public health question of susceptibility in the general population. Just 1 in 5 patients with COVID-19 are admitted to hospital, therefore studies in these settings are not well placed to assess susceptibility in the community (16). Furthermore, there was limited adjustment for confounders in some of these studies and the source populations in some studies included patients without diabetes (14, 15). Only 2 papers included any propensity score matching to mitigate the effect of prescription by indication bias (17, 18). None of the studies considered unobserved confounders.

Mechanistically, metformin is associated with adenosine monophosphate–activated protein kinase (AMPK) activation. AMPK has been shown to both increase the expression of the angiotensin-converting enzyme 2 (ACE2) receptor, and to increase stability of the ACE2 receptor via ACE2 Ser680 phosphorylation in human cells in vitro (19). It has been suggested that this phosphorylation of ACE2 receptor may lead to decreased binding with COVID-19 due to conformational and functional change in the receptor (20). Others, however, have argued that this increased ACE2 receptor stability may be associated with an increase in susceptibility to infection (21).

Current “sick-day rules” guidelines advise metformin should be discontinued by patients hospitalized for acute illnesses (22). Metformin may increase the risk of lactic acidosis or acute kidney injury in severe COVID-19 (18), especially if it exacerbates gastrointestinal symptoms (22). This guidance has been reiterated by National Health Service (NHS) England for COVID-19 (23).

Given the proposed mechanisms by which metformin might impact susceptibility to COVID-19, we aimed to investigate in a primary care–based type 2 diabetes patient cohort the risk of confirmed or suspected COVID-19 with current metformin prescription to those with a history or absence of a metformin prescription.

## Methods

### Study Design

A population-based retrospective cohort study of adults with type 2 diabetes comparing those with current prescription of metformin and at least another glucose-lowering agent (MF+) with those with a current prescription of other glucose-lowering agents that does not include metformin (MF−). Data source and methods are broadly similar to our recent publication investigating the susceptibility to COVID-19 in patients prescribed with sodium-glucose cotransporter 2 (SGLT2) in comparison with dipeptidyl peptidase 4 (DPP4) inhibitors (24).

### Data Source

The data for this cohort study was derived from The Health Improvement Network (THIN), which contains computerized primary records covering approximately 2.1 million active participants from 365 general practices that used Vision electronic medical records software across the UK in 2020 (25). In terms of demographic structure and common morbidity prevalence, THIN has been demonstrated to be representative of the UK population (25). Within THIN, information relating to symptoms, examinations, investigations, and diagnoses are recorded as Read codes, a hierarchical clinical coding system that is updated in response to new medical knowledge and requests from clinicians (26). Prescriptions are recorded based on the dictionary of medicines and devices (dm + d) and Anatomical Therapeutic Classification (ATC) systems (27). To improve data quality, general practices were eligible to be included in this study from the later of 12 months after the installation of Vision software and 12 months after reporting acceptable mortality rates (28).

### Study Population

Adults (≥18 years of age) with a record of type 2 diabetes and registered with an eligible general practice no later than January 30, 2019 (1 year before the index date) were eligible for this study. Any individual who was diagnosed with diabetes before the age of 12 years, with a record of type 1 diabetes, adverse reaction to glucose-lowering agents, pancreatitis, estimated glomerular filtration rate (eGFR) measurement less than 30mL/min/1.73m^2^ before the index date, or pregnancy in the preceding year, was excluded. Type 2 diabetes were identified by Read code diagnoses (Supplementary file S1) (30), which have been previously validated within this dataset (29, 30).

### Definitions of Glucose-Lowering Agents

Glucose-lowering agent users were defined as individuals with a record of 1 of the following 9 prescriptions: metformin, SGLT2 inhibitors, DPP4 inhibitors, glucagon-like peptide-1 (GLP-1), insulin, sulfonylureas, meglitinides, thiazolidinedione, and acarbose. For each glucose-lowering agent, participants were classified into 4 mutually exclusive categories: current users (prescription continuing beyond the index date), recent users (prescription within 90 days preceding the index date), historical users (prescription ended at least 90 days preceding the index date), and nonusers.

### Inclusion and Exclusion Criteria for Metformin Cohort (Exposed Cohort)

To be included in the exposed cohort (MF+), patients should be on a current metformin prescription (extending into the pandemic period) and co-prescribed at least 1 of 8 other glucose-lowering agents. Patients who were on metformin monotherapy were excluded as they would be at an early disease status, resulting in immortal time bias (31).

### Inclusion and Exclusion Criteria for Comparator Medication Cohort (Unexposed Cohort)

To be included in the unexposed cohort (MF−), patients should not be a current or recent user of metformin and should have a prescription for at least 1 of the 8 other glucose-lowering agents extending into the pandemic period.

### Matching

Individuals in the exposed groups were propensity score–matched to individuals in the comparator group. Variables used for estimating propensity score for use of metformin (MF+) included: sociodemographic characteristics including age and sex; lifestyle and metabolic profile measures including body mass index (BMI), smoking status, alcoholism, systolic and diastolic blood pressure, total cholesterol, high-density lipoprotein cholesterol (HDL-C), albumin-creatinine ratio (ACR), eGFR, and glycated hemoglobin (HbA1c); presence of comorbidities, including atrial fibrillation, hypertension, cardiovascular diseases (ischemic heart disease, heart failure, stroke/transient ischemic attack, and peripheral vascular disease), and rheumatoid arthritis; comorbidities identified as risk factors for COVID-19, including respiratory diseases, cancers, and rare metabolic disorders; diabetes complications or measures indicating diabetes severity, including microvascular conditions (peripheral neuropathy, diabetes-related foot disease, sight-threatening retinopathy); diabetes duration; and history of prescriptions of relevant drugs including renin-angiotensin-aldosterone system (RAAS) inhibitors, other antihypertensive drugs, lipid-lowering drugs, antiplatelets, anticoagulants, immunosuppressant therapies (including oral corticosteroids), and prescription status of individual glucose-lowering agent (SGLT2 inhibitors, DPP4 inhibitors, GLP-1, insulin, sulfonylureas, meglitinides, thiazolidinedione, and acarbose). Multiple imputation by groups method was used for missing data of all continuous variables (BMI, systolic and diastolic blood pressure, total cholesterol, HDL-C, albumin-creatinine ratio, eGFR, and HbA1c), with 5 simulated complete datasets being created, under the assumption of missing at random. For each simulated complete dataset, individuals in the exposed group were then matched 1:1 to individuals in the comparator group by propensity score using the nearest neighbor algorithm. To ensure positivity (having adequate variation in the treatment of interest within confounder strata), propensity scores were truncated and only participants with propensity scores that lay on the common support with a caliper width of 0.2 were eligible for matching (32). Following matching, each of the imputed propensity score–matched dataset was independently analyzed by standard methods and the results combined in a straightforward manner to produce estimates and CIs that incorporate missing-data uncertainty, using the Rubin rules.

### Follow-Up Period

Individuals were followed up from January 30, 2020 (index date) until the earliest date of the following events: outcome of interest, death, individual left practice, practice ceased contributing to the database, or study end date (October 13, 2020).

### Outcome

Outcomes were composite of confirmed and clinically suspected diagnosis of COVID-19, confirmed COVID-19, and COVID-19-related deaths, and all-cause mortality. COVID-19-related death was defined as individuals who died within 28 days after the diagnosis of suspected or confirmed COVID-19. A negative control outcome—incident back pain (a common presentation in primary care)—during follow-up was used to evaluate the possibility of surveillance bias and unobserved confounding bias (33). We chose back pain as a negative outcome, considering it is a common presentation in primary care and is not associated with the exposure or outcome of interest. All outcomes were defined by the relevant clinical (Read) codes (Supplementary file S2) (30). In the UK, positive reverse transcription polymerase chain reaction (RT-PCR) swab results or positive antibody tests are defined as confirmed COVID-19. Clinicians entered a suspected COVID-19 code where there was no RT-PCR/antibody testing available but a clinical picture was consistent with this infection, confirmation with other investigations (eg, imaging) and/or the patient had been in contact with a confirmed case.

### Covariates

The covariate data recorded prior to the index date were obtained and used for propensity score matching and adjustment in the outcome model. Individuals’ BMIs were classified using World Health Organization criteria as follows: underweight or normal weight (BMI of <25 kg/m^2^), overweight (BMI of 25 kg/m^2^ to <30 kg/m^2^), and obesity (BMI of ≥30 kg/m^2^). Smoking status was classified as nonsmokers, ex-smokers, or current smokers. Comorbid conditions and diabetes complications were defined by coded diagnoses (Read codes) recorded in THIN. Comorbid conditions included atrial fibrillation, hypertension, cardiovascular diseases (ischemic heart disease, heart failure, stroke/transient ischemic attack, and peripheral vascular disease), rheumatoid arthritis, respiratory diseases, cancers, nonalcoholic fatty liver disease or nonalcoholic steatohepatitis (NAFLD/NASH), and rare metabolic disorders. Physiological/laboratory measurements were categorized based on clinically meaningful thresholds. Missing data for smoking status and physiological/laboratory measurements were treated as a separate missing category for each variable. The absence of a record of any diagnosis or prescription was taken to indicate the absence of these conditions or medications, respectively.

### Statistical Analysis

Baseline characteristics of individuals in the exposed and comparator groups were reported using appropriate descriptive statistics (mean for normally distributed continuous variables and proportions for categorical variables) before and after propensity score matching. Histograms of propensity scores were generated before and after matching for a visual check of the global balance of propensity scores between groups.

Crude incidence rate per 1000 person-years of the outcome of interest and the negative control outcome were estimated for the exposed and the comparator groups. Cox proportional hazards regression models were used to calculate the crude and adjusted hazard ratios, together with their corresponding 95% CIs. Other than rare metabolic conditions (excluded due to the limited number), systolic and diastolic blood pressure (to avoid double adjustment alongside hypertension diagnosis), and prescription status of glucose-lowering agent, all variables listed for propensity score matching and NAFLD/NASH were included in the Cox models for the unmatched analyses. Additionally, we used a composite of cardiovascular conditions and a composite of diabetes complications instead of their individual components in the Cox model adjustment. Survival curves of exposed and comparator groups were generated for both the unmatched and the propensity score–matched cohorts.

A sensitivity analysis limiting the unexposed cohort to those who have previously used metformin but discontinued was performed to eliminate the potential selection bias induced by patients who have not been prescribed metformin before. As metformin might have different performance in different populations, subgroup analyses were also conducted in female, male, individuals with a BMI value ≥ 25 kg/m^2^, and those with a BMI value ≥ 30kg/m^2^, respectively.

## Results

**Figure 1.**
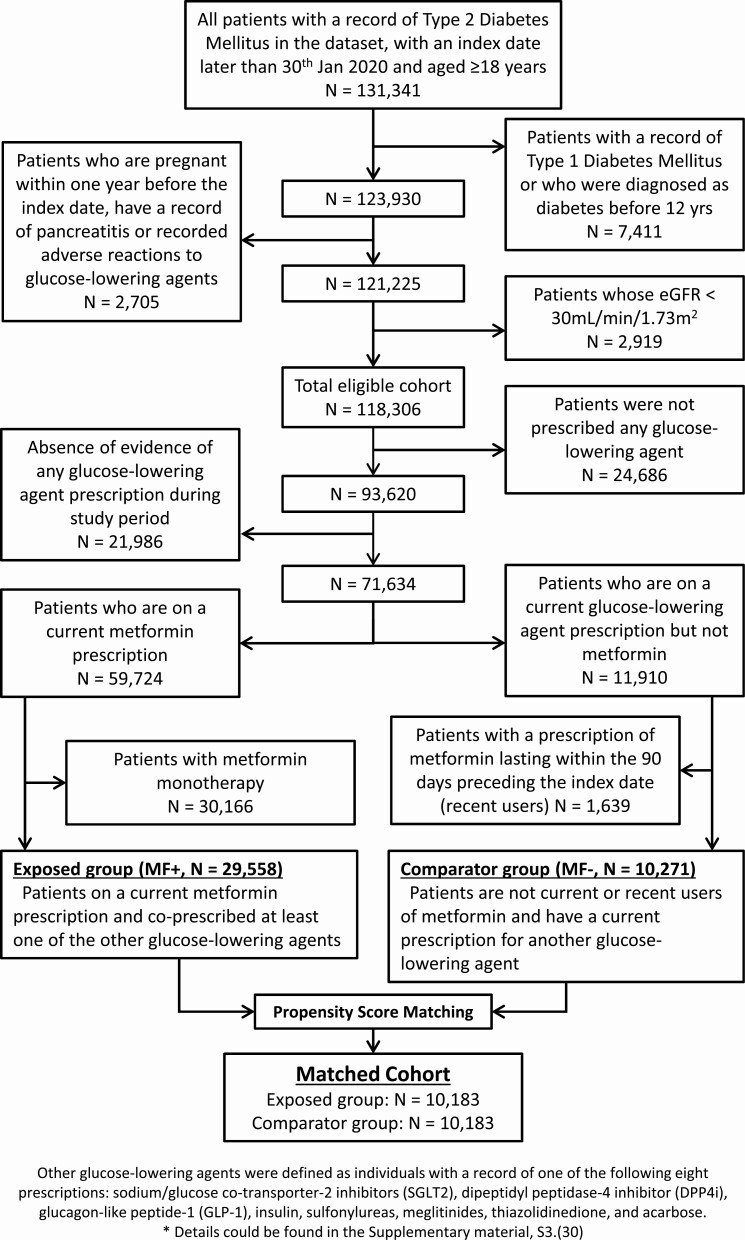
Flow chart.

There were 29 558 individuals with a current prescription of metformin (MF+) (exposed group) and 10 271 individuals with a current prescription of other glucose-lowering agents (MF−) (comparator group). Figure 1 shows the flow chart of participant selection procedure for this study.

### Baseline Characteristics

In the unmatched cohort, individuals with a current prescription of metformin with other glucose-lowering agents (MF+) were younger (mean age 64.8 vs 67.8 years), had a greater proportion of males (61.9% vs 52.0%), slightly higher BMI value (mean BMI 32.1 vs 31.8kg/m^2^), and a lower prevalence of comorbidities, and longer mean duration of diabetes (12.1 vs 11.6 years), compared with individuals with a current prescription that does not include metformin (MF− comparator group) (Table 1).

**Table 1.** Baseline Demographic Characteristics, Behavioral Risk Factors, Diabetes Complications, Comorbidities, Metabolic Characteristics, and Medications

	Unmatched		Propensity score–matched^*b*^	
	Exposed group (MF+) (n = 29 558)	Comparator group (MF−) (n = 10 271)	Exposed group (MF+) (n = 10 183)	Comparator group (MF−) (n = 10 183)
***Demographic characteristics***				
Mean age, years (SD)	64.8 (11.7)	67.8 (13.1)	67.2 (11.8)	67.7 (13.1)
Male sex (%)	18 298 (61.9)	5343 (52.0)	5297 (52.0)	5326 (52.3)
Mean BMI, kg/m^2^ (SD)	32.1 (6.70)	31.8 (6.8)	31.9 (6.7)	31.8 (6.8)
**BMI category (%)**				
<25	3159 (10.7)	1353 (13.2)	1199 (11.8)	1338 (13.1)
25 to <30	9080 (30.7)	3067 (29.9)	3084 (30.3)	3038 (29.8)
≥30	16 908 (57.2)	5678 (55.3)	5706 (56.0)	5639 (55.4)
Missing	411 (1.4)	173 (1.7)	194 (1.9)	168 (1.6)
***Behavioral risk factors***				
**Smoking status (%)**				
Nonsmokers	14 632 (49.5)	5105 (49.7)	4997 (49.1)	5057 (49.7)
Ex-smokers	10 799 (36.5)	3726 (36.3)	3710 (36.4)	3701 (36.3)
Current smokers	4063 (13.7)	1383 (13.5)	1432 (14.1)	1373 (13.5)
Missing	64 (0.2)	57 (0.6)	44 (0.4)	52 (0.5)
Excessive alcohol use (%)	1506 (5.1)	668 (6.5)	655 (6.4)	657 (6.5)
Mean diabetes duration, years (SD)	12.1 (6.7)	11.6 (7.0)	11.6 (6.7)	11.6 (7.0)
***Diabetes complications***				
Peripheral neuropathy (%)	2070 (7.0)	784 (7.6)	775 (7.6)	776 (7.6)
Diabetic foot disease (%)	1335 (4.5)	527 (5.1)	526 (5.2)	518 (5.1)
Sight threating retinopathy (%)	3507 (11.9)	1286 (12.5)	1244 (12.2)	1264 (12.4)
***Comorbidities***				
Hypertension (%)	17 477 (59.1)	6234 (60.7)	6091 (59.8)	6178 (60.7)
Ischemic heart disease (%)	5374 (18.2)	2271 (22.1)	2216 (21.8)	2237 (22.0)
Atrial fibrillation (%)	2194 (7.4)	1101 (10.7)	1037 (10.2)	1077 (10.6)
Stroke/TIA (%)	1992 (6.7)	1001 (9.7)	944 (9.3)	970 (9.5)
Heart failure (%)	1322 (4.5)	764 (7.4)	696 (6.8)	745 (7.3)
Peripheral vascular disease (%)	1055 (3.6)	508 (4.9)	492 (4.8)	500 (4.9)
Chronic kidney disease (%)	3839 (13.0)	2481 (24.2)	1842 (18.1)	2428 (23.8)
Cancers^*a*^ (%)	3114 (10.5)	1505 (14.7)	1481 (14.5)	1474 (14.5)
Chronic respiratory disease (%)	2351 (8.0)	1031 (10.0)	1009 (9.9)	1016 (10.0)
Liver disease (%)	1636 (5.5)	624 (6.1)	570 (5.6)	619 (6.1)
Rheumatoid arthritis (%)	442 (1.5)	218 (2.1)	220 (2.2)	212 (2.1)
NAFLD/NASH (%)	742 (2.5)	263 (2.6)	262 (2.6)	261 (2.6)
Blood & bone marrow cancer (%)	404 (1.4)	163 (1.6)	165 (1.6)	162 (1.6)
***Metabolic characteristics***				
**Diastolic BP (mmHg)**				
<90	27 474 (92.9)	9530 (92.8)	9478 (93.1)	9450 (92.8)
≥90	2052 (6.9)	727 (7.1)	689 (6.8)	720 (7.1)
Missing	32 (0.1)	14 (0.1)	16 (0.2)	13 (0.1)
**Systolic BP (mmHg)**				
<140	21 102 (71.4)	7120 (69.3)	7078 (69.5)	7073 (69.5)
≥140	8424 (28.5)	3137 (30.5)	3089 (30.3)	3097 (30.4)
Missing	32 (0.1)	14 (0.1)	16 (0.2)	13 (0.1)
**Total cholesterol (mmol/L)**				
<5.2	25 257 (85.4)	8106 (78.9)	8137 (79.9)	8064 (79.2)
5.2 to 6.2	2878 (9.7)	1388 (13.5)	1251 (12.3)	1367 (13.4)
≥6.2	1252 (4.2)	696 (6.8)	725 (7.1)	675 (6.6)
Missing	171 (0.6)	81 (0.8)	70 (0.7)	77 (0.8)
**HDL-cholesterol (mmol/L)**				
<1.55	26 054 (88.1)	8736 (85.1)	8757 (86.0)	8669 (85.1)
≥1.55	3028 (10.2)	1312 (12.8)	1239 (12.2)	1296 (12.7)
Missing	476 (1.6)	223 (2.2)	187 (1.8)	218 (2.1)
**eGFR (mL/min/1.73m** ^**2**^)				
≥60 (Stage 1&2)	24 469 (82.8)	7280 (70.9)	7597 (74.6)	7258 (71.3)
30 to 60 (Stage 3)	4991 (16.9)	2950 (28.7)	2551 (25.1)	2885 (28.3)
Missing	98 (0.3)	41 (0.4)	35 (0.3)	40 (0.4)
**ACR (mg/mmol)**				
<3 mg/mmol	15 119 (51.2)	4851 (47.2)	4909 (48.2)	4819 (47.3)
3 to 30 mg/mmol	6144 (20.8)	2274 (22.1)	2294 (22.5)	2258 (22.2)
≥30 mg/mmol	1052 (3.6)	481 (4.7)	460 (4.5)	470 (4.6)
Missing	7243 (24.5)	2665 (25.9)	2520 (24.7)	2636 (25.9)
**HbA1c (mmol/mol)**				
<48 mmol/mol (6.5%)	2654 (9.0)	1048 (10.2)	879 (8.6)	1042 (10.2)
48 to 58 mmol/mol (6.5–7.5%)	7918 (26.8)	2645 (25.8)	2618 (25.7)	2624 (25.8)
≥58 mmol/mol (7.5%)	18 461 (62.5)	6429 (62.6)	6489 (63.7)	6369 (62.5)
Missing	525 (1.8)	149 (1.5)	197 (1.9)	148 (1.5)
***Medications***				
**Metformin (%)**				
Current users	29 558 (100.0)	0 (0.0)	10 183 (100.0)	0 (0.0)
Recent users	0 (0.0)	0 (0.0)	0 (0.0)	0 (0.0)
Historical users	0 (0.0)	8640 (84.1)	0 (0.0)	8587 (84.3)
Nonusers	0 (0.0)	1631 (15.9)	0 (0.0)	1596 (15.7)
**DPP4i (%)**				
Current users	13 132 (44.4)	4249 (41.4)	4170 (41.0)	4222 (41.5)
Recent users	364 (1.2)	147 (1.4)	138 (1.4)	146 (1.4)
Historical users	4985 (16.9)	1809 (17.6)	1773 (17.4)	1795 (17.6)
Nonusers	11 077 (37.5)	4066 (39.6)	4102 (40.3)	4020 (39.5)
**SGLT2i (%)**				
Current users	10 661 (36.1)	2784 (27.1)	2871 (28.2)	2780 (27.3)
Recent users	278 (0.9)	103 (1.0)	97 (1.0)	102 (1.0)
Historical users	2055 (7.0)	844 (8.2)	855 (8.4)	836 (8.2)
Nonusers	16 564 (56.0)	6540 (63.7)	6360 (62.5)	6465 (63.5)
**Meglitinides (%)**				
Current users	60 (0.2)	13 (0.1)	10 (0.1)	13 (0.1)
Recent users	6 (0.0)	0 (0.0)	0 (0.0)	0 (0.0)
Historical users	306 (1.0)	121 (1.2)	121 (1.2)	118 (1.2)
Nonusers	29 186 (98.7)	10 137 (98.7)	10 052 (98.7)	10 052 (98.7)
**Acarbose (%)**				
Current users	42 (0.1)	15 (0.1)	15 (0.1)	15 (0.1)
Recent users	4 (0.0)	1 (0.0)	1 (0.0)	1 (0.0)
Historical users	219 (0.7)	105 (1.0)	112 (1.1)	103 (1.0)
Nonusers	29 293 (99.1)	10 150 (98.8)	10 055 (98.7)	10 064 (98.8)
**GLP1 (%)**				
Current users	2081 (7.0)	588 (5.7)	618 (6.1)	586 (5.8)
Recent users	597 (2.0)	157 (1.5)	160 (1.6)	156 (1.5)
Historical users	2910 (9.8)	960 (9.3)	1018 (10.0)	954 (9.4)
Nonusers	23 970 (81.1)	8566 (83.4)	8387 (82.4)	8487 (83.3)
**Insulin (%)**				
Current users	1901 (6.4)	1029 (10.0)	1005 (9.9)	1006 (9.9)
Recent users	859 (2.9)	361 (3.5)	358 (3.5)	360 (3.5)
Historical users	2613 (8.8)	1193 (11.6)	1149 (11.3)	1169 (11.5)
Nonusers	24 185 (81.8)	7688 (74.9)	7671 (75.3)	7648 (75.1)
**Thiazolidinedione (%)**				
Current users	1683 (5.7)	547 (5.3)	541 (5.3)	542 (5.3)
Recent users	69 (0.2)	32 (0.3)	34 (0.3)	32 (0.3)
Historical users	4422 (15.0)	1404 (13.7)	1437 (14.1)	1388 (13.6)
Nonusers	23 384 (79.1)	8288 (80.7)	8171 (80.2)	8221 (80.7)
**Sulphonylureas (%)**				
Current users	12 979 (43.9)	4510 (43.9)	4457 (43.8)	4473 (43.9)
Recent users	565 (1.9)	233 (2.3)	226 (2.2)	231 (2.3)
Historical users	6363 (21.5)	2523 (24.6)	2479 (24.3)	2491 (24.5)
Nonusers	9651 (32.7)	3005 (29.3)	3021 (29.7)	2988 (29.3)
**Immunosuppressive drugs (%)**	1070 (3.6)	592 (5.8)	548 (5.4)	578 (5.7)
**Systemic corticosteroids (%)**	3047 (10.3)	1366 (13.3)	1352 (13.3)	1354 (13.3)
**ACEi/ARB (%)**	21 667 (73.3)	7380 (71.9)	7293 (71.6)	7316 (71.8)
**Other antihypertensives (%)**	19 799 (67.0)	7408 (72.1)	7301 (71.7)	7337 (72.1)
**Anticoagulants (%)**	3628 (12.3)	1768 (17.2)	1677 (16.5)	1731 (17.0)
**Antiplatelets (%)**	14 988 (50.7)	5471 (53.3)	5354 (52.6)	5411 (53.1)
**Lipid-lowering drugs (%)**	26 074 (88.2)	8748 (85.2)	8711 (85.5)	8686 (85.3)

Exposed group: patients with a current combination prescription of metformin and other glucose-lowering agents; Comparator group: patients with a current prescription of other glucose-lowering agents

Abbreviations: ACEi, angiotensin-converting enzyme inhibitor; ACR, albumin-creatinine ratio; ARB, angiotensin receptor blocker; BMI, body mass index; BP, blood pressure; DPP4i, dipeptidyl peptidase-4 inhibitors; eGFR, estimated glomerular filtration rate; GLP1, glucagon-like peptide 1 receptor agonist; HbA1c, glycated hemoglobin A1c; HDL, high-density lipoprotein; NAFLD/NASH, nonalcoholic fatty liver disease/nonalcoholic steatohepatitis; SGLT2i, sodium-glucose cotransporter-2 inhibitors; TIA, transient ischemic attack.

^a^Excluding melanoma, and blood and bone marrow cancers

^*b*^ Data were presented for the first multiple imputed propensity score–matched cohort.

Following propensity score matching, 10 183 individuals of the metformin group (MF+) were compared with 10 183 individuals in the comparator group (MF−). The baseline characteristics of the exposed (MF+) and comparator (MF−) groups, including demographic and behavioral risk factors, diabetes duration, diabetes complications, comorbidities, metabolic profile, and prior prescriptions, were generally similar in all 5 imputed propensity score–matched datasets (Table 1, Supplementary file S4) (30). Although individuals in the exposed group (MF+) still had a lower prevalence of chronic kidney disease (18.1% vs 23.8% in the first imputed propensity score–matched datasets) compared with the comparator group (MF−), the imbalance between the groups from the unmatched cohort was largely mitigated.

### Suspected or Confirmed COVID-19

There were 415 individuals (110 were confirmed COVID-19) in the exposed group (MF+) and 188 individuals (54 were confirmed COVID-19) in the comparator group (MF−) who were diagnosed with suspected/confirmed COVID-19, corresponding to a crude incidence rate of 20.4 and 26.9 per 1000 person-years, respectively.

**Figure 2.**
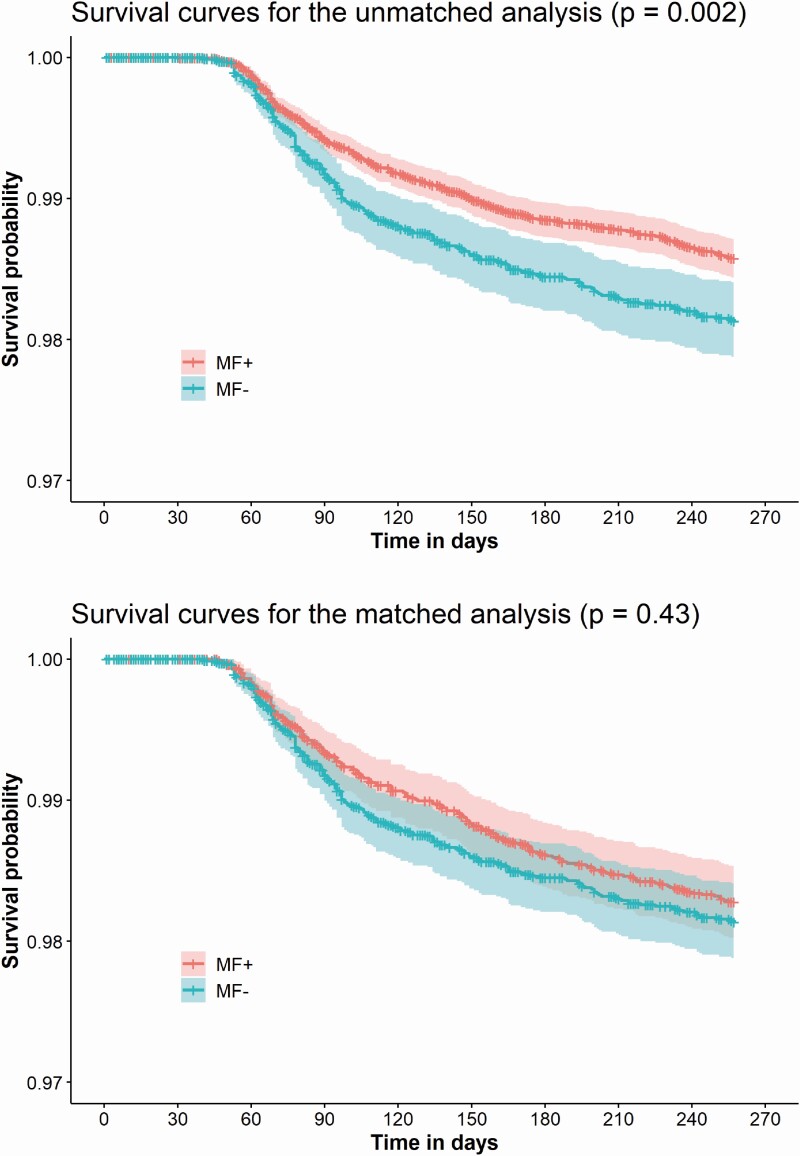
Kaplan-Meier plot showing the risk of suspected/confirmed COVID-19 in the unmatched and matched analysis.

Following propensity score matching, 172 individuals (47 had confirmed COVID-19) in the exposed group (MF+) and 186 individuals (53 had confirmed COVID-19) in the comparator group were diagnosed with suspected/confirmed COVID-19, corresponding to a crude incidence rate of 24.7 and 26.8 per 1000 person-years, respectively. In the exposed group (MF+) compared to the comparator group (MF−), the adjusted hazard ratio (HR) for primary care consultations for suspected/confirmed COVID-19 was 0.85 (95% CI, 0.67-1.08) and for confirmed COVID-19 was 0.80 (95% CI, 0.49-1.30) (Table 2). Kaplan-Meier curves for suspected/confirmed COVID-19 cases are presented in Fig. 2.

**Table 2.** Risk of Developing COVID-19, Mortality, and Back Pain Among Those With a Current Combination Prescription of Metformin and Other Glucose-Lowering Agents Compared With a Current Prescription of Other Glucose-Lowering Agents Only

	Confirmed COVID-19		Suspected/Confirmed COVID-19		COVID-19-related death		All-cause mortality		Back pain	
	Exposed group (MF+)	Comparator group (MF−)	Exposed group (MF+)	Comparator group (MF−)	Exposed group (MF+)	Comparator group (MF−)	Exposed group (MF+)	Comparator group (MF−)	Exposed group (MF+)	Comparator group (MF−)
**Unmatched analysis**										
Total number of patients	29 558	10 271	29 558	10 271	29 558	10 271	29 558	10 271	29 558	10 271
Number of outcomes	110	54	415	188	31	21	403	275	315	109
Person-years (py)	20 454	7038	20 351	6993	20 486	7055	20 486	7055	20 364	7010
Incidence rate (per 1000 py)	5.4	7.7	20.4	26.9	1.5	3.0	19.7	39.0	15.5	15.6
Crude hazard ratio (95% CI)	0.70 (0.51, 0.97)		0.76 (0.64, 0.90)		0.51 (0.29, 0.89)		0.51 (0.43, 0.59)		1.00 (0.80, 1.24)	
Adjusted HR (95% CI)^*a*^	0.75 (0.54, 1.04)		0.80 (0.67, 0.96)		0.74 (0.42, 1.32)		0.72 (0.62, 0.85)		1.01 (0.81, 1.26)	
**Matched analysis**										
Total number of patients^*b*^	10 183	10 183	10 183	10 183	10 183	10 183	10 183	10 183	10 183	10 183
Number of outcomes^*b*^	47	53	172	186	17	20	214	266	107	108
Person-years (py) ^*b*^	7010	6980	6969	6935	7023	6998	7023	6998	6981	6952
Incidence rate (per 1000 py) ^*b*^	6.7	7.6	24.7	26.8	2.4	2.9	30.5	38.0	15.3	15.5
Crude hazard ratio (95% CI)^ǂ^	0.78 (0.48, 1.28)		0.85 (0.67, 1.08)		0.76 (0.29, 1.97)		0.79 (0.66, 0.95)		1.01 (0.73, 1.39)	
Adjusted HR (95% CI) ^*ab*^	0.80 (0.49, 1.30)		0.85 (0.67, 1.08)		0.87 (0.34, 2.20)		0.89 (0.74, 1.07)		1.01 (0.73, 1.39)	

Exposed group: patients with a current combination prescription of metformin and other glucose-lowering agents; Comparator group: patients with a current prescription of other glucose-lowering agents

^*a*^Adjusted for: age, sex, smoking status, high alcohol consumption (alcoholism), body mass index categories, total cholesterol categories, high-density lipoprotein categories, albumin-creatinine ratio categories, estimated glomerular filtration rate categories, HbA1c categories, atrial fibrillation, rheumatoid arthritis, hypertension, cardiovascular disease, nonalcoholic fatty liver disease or nonalcoholic steatohepatitis, prescriptions of renin-angiotensin-aldosterone system inhibitors, other antihypertensive drugs, lipid-lowering drugs, antiplatelets, anticoagulants, diabetes complications, diabetes duration, respiratory disease, cancers, immunosuppressant therapies, and systemic corticosteroid use.

^*b*^Data were presented for the first multiple imputed propensity score–matched cohort.

### All-Cause Mortality and COVID-19-Related Death

During the study period there were 403 deaths (31 were COVID-19-related deaths) in the exposed group (MF+) and 275 (21 were COVID-19-related deaths) in the comparator group (MF−), corresponding to a crude incidence rate of 19.7 and 39.0 per 1000 person-years, respectively.

Following propensity score matching, 17 of 214 deaths in the exposed group (MF+) and 20 of 266 deaths in the comparator group MF−) were related to COVID-19. No statistically significant difference was observed between the groups for the risk of all-cause mortality (adjusted HR 0.89; 95% CI, 0.74-1.07) or COVID-19-related death (adjusted HR 0.87; 95% CI, 0.34-2.20).

### Back Pain (Negative Outcome)

Among individuals who were free of back pain at study entry, there were 119 and 51 individuals in the exposed (MF+) and the comparator group (MF−) diagnosed with back pain during the follow-up period, respectively.

In the propensity score–matched cohort, 107 individuals in the exposed group (MF+) and 108 individuals in the comparator group were patients with incident back pain, which translated to an adjusted HR of 1.01 (95% CI, 0.73-1.39).

The results were broadly similar in the sensitivity analyses as well as the subgroup analyses (Supplementary files S5-S7) (30).

## Discussion

Type 2 diabetes has been identified as a predictor of severe COVID-19 and mortality (3, 34, 35). However, little is currently known about which specific glucose-lowering agents can be used to safely maintain or improve glycemic control amid the COVID-19 pandemic (3, 36). In our primary care–based study, we found that patients with type 2 diabetes who received a prescription of metformin (plus other glucose-lowering agents) within 90 days of the study start period were not more likely to present to primary care with confirmed or suspected COVID-19-related disease compared with those not taking metformin. There was no significant increase in COVID-19-related mortality or all-cause mortality.

### Relationship to Other Studies

**Figure 3.**
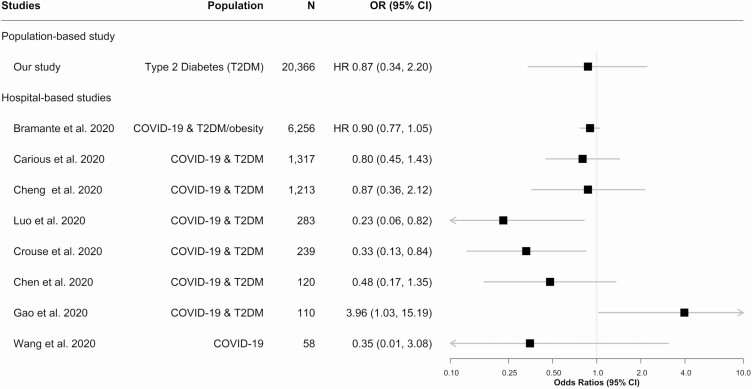
Forest plots showing the results of previous similar studies on the association between metformin use and COVID-19-related mortality.

There are currently no comparable studies in the published or preprint literature investigating the association between therapeutic prescription of metformin and primary care consultation for symptomatic COVID-19. Several observational studies have examined the association between mortality from COVID-19 and taking metformin in more severely affected hospitalized patients and have suggested a reduction in risk (15, 16). Our findings in primary care are in line with those reported by Bramante et al from a propensity score–matched observational study of mortality in over 6000 US patients hospitalized with COVID-19, which found that metformin had no effect on in-hospital mortality in patients with type 2 diabetes or obesity (17). However, Bramante et al found lower in-hospital mortality with COVID-19 in women taking metformin (OR 0.76; 95% CI, 0.60-0.96), while there was no significant reduction in mortality for men (17). In our study, we found susceptibility to COVID-19 was statistically significant lower (adjusted HR 0.68; 95% CI, 0.48-0.96) in women taking metformin, compared with those taking other glucose-lowering agents. The results of previous relevant studies and our study on the association between metformin use and COVID-19-related mortality have been shown in Fig. 3 (4, 7, 17, 18, 37-40).

The pathobiology of any interaction between COVID-19 and metformin is incompletely understood but may be affected by factors that impact the ability of the virus to enter host cells, the ability of the host to clear the virus through disease resistance mechanisms, and host ability to withstand a deterioration in organ function through disease tolerance mechanisms (41). Firstly, metformin may prevent or reduce the SARS-CoV-2 virus from entering cells (20). It may decrease potentially harmful levels of proinflammatory cytokines which drive hyperinflammation through macrophage M1 to M2 class switching (42-44). Furthermore, metformin has been shown to potentially inhibit checkpoint inhibitor upregulation which may be important to avoid T-cell anergy and promote clearance of SARS-CoV-2 (44, 45). However, the AMPK-mediated immunomodulatory effects of metformin via the antiviral interferon pathway have also been noted to impair antibody responses following vaccination in people with diabetes so that the net effect of metformin in terms of propensity to develop or die from COVID-19 is neutral (46), as we have found.

### Strengths and Limitations

Our study included a large cohort and the study period covers the majority of the pandemic duration in the UK to date. The proportion of missing variables was low. We used a propensity score–matched design in order to minimize any effects due to differences between the exposed and unexposed groups, due to or confounding by indication bias. Furthermore, we adjusted for a range of potential confounders and included a negative control outcome in our analyses.

However, there are several important limitations. The data quality is reliant on accurate coding by general practitioners and administrative staff in primary care, including coding of hospital discharge letters sent to primary care services for patients with suspected or confirmed COVID-19. In the UK, all patients with type 2 diabetes are entitled to free and easily accessible prescription of glucose-lowering agents (prescription-only medications) from their general practitioner. Only an extremely small proportion of patients would receive their care through the private sector, therefore this would not have a significant impact on the generalizability of our results. Recording of medication prescriptions is known to be very reliable in this primary care dataset, with very few patients not accessing them via their NHS general practitioner. However, it is possible that patients are not compliant with their medication. The event rate for some outcomes, such as confirmed COVID-19 cases and COVID-19-related deaths, was relatively low, limiting statistical power to detect small effects in these outcomes. Although the groups were well matched for multiple factors that could affect the outcome, there remained some imbalance with respect to eGFR/chronic kidney disease status, which is known to impact COVID-19 outcomes (34); however, eGFR was adjusted for in the regression models. We did not have data on hospitalization status, patient ethnicity, or socioeconomic status. It is not possible to completely rule out unmeasured confounding.

Although we found no significant harm or benefit of metformin, the study was not designed to assess the effects of metformin on COVID-19 outcomes in patients who do not have type 2 diabetes. Consequently, it remains unclear whether metformin could benefit any specific groups of patients without diabetes (eg, those with obesity, or prediabetes) in the context of COVID-19.

### Recommendations for Primary Care

Our findings show that metformin did not significantly influence susceptibility to COVID-19 no mortality from it among people with type 2 diabetes in the UK primary care setting. This is important, as a majority of patients with type 2 diabetes are prescribed metformin (47). For many patients, metformin is crucial to optimizing glycemic control and weight management, and it has a long-established efficacy and safety profile (48). Our data, in conjunction with those from hospitalized patients, demonstrate that patients who are prescribed long-term metformin and are currently well can be reassured that it is safe to continue consumption. Adherence to usual guidance on sick-day rules, however, remains strongly advised if the patient is at risk of dehydration (22, 23). Clinicians should advise patients taking metformin who develop symptoms consistent with COVID-19 (or indeed any infection) to withhold this medication if there are concerns about acidosis and to consider increased monitoring of blood glucose for the duration of the illness (23).

## Conclusion

Our findings suggest that prescription of metformin in primary care does not influence susceptibility to COVID-19, COVID-19-related mortality, or all-cause mortality. This is reassuring given that patients with diabetes are more susceptible to mortality with COVID-19, and that metformin is the most commonly prescribed glucose-lowering medication. Optimizing glycemic control should continue to be the best advice for patients with diabetes, especially if rates of COVID-19 rise.

## Data Availability

The datasets generated during and/or analyzed during the current study are not publicly available but are available from the corresponding author on reasonable request.

## Abbreviations

ACE2: angiotensin converting enzyme 2
AMPK: adenosine monophosphate–activated protein kinase
BMI: body mass index
DPP4: dipeptidyl peptidase 4
eGFR: estimated glomerular filtration rate
GLP-1: glucagon-like peptide-1
HbA1c: glycated hemoglobin
HDL-C: high-density lipoprotein cholesterol
NAFLD: nonalcoholic fatty liver disease
NASH: nonalcoholic steatohepatitis
SGLT2: sodium-glucose cotransporter 2
THIN: The Health Improvement Network

## Additional Information

***Disclosures:*** The authors have no conflicts of interest relevant to this article to disclose.
